# Preclinical characterization and phase I clinical trial of CT053PTSA targets MET, AXL, and VEGFR2 in patients with advanced solid tumors

**DOI:** 10.3389/fimmu.2022.1024755

**Published:** 2022-10-20

**Authors:** Yu-Xiang Ma, Fu-Rong Liu, Yang Zhang, Qun Chen, Zhi-Qiang Chen, Qian-Wen Liu, Yan Huang, Yun-Peng Yang, Wen-Feng Fang, Ning Xi, Ning Kang, Yu-Lei Zhuang, Qi Zhang, Ying-Zhi Jiang, Li Zhang, Hong-Yun Zhao

**Affiliations:** ^1^ Department of Clinical Research, State Key Laboratory of Oncology in South China, Collaborative Innovation Center for Cancer Medicine, Sun Yat-Sen University Cancer Center, Guangzhou, China; ^2^ Department of Medical Oncology, State Key Laboratory of Oncology in South China, Collaborative Innovation Center for Cancer Medicine, Sun Yat-Sen University Cancer Center, Guangzhou, China; ^3^ HEC R&D Center, Sunshine Lake Pharma Co., Ltd, Donggguan, China

**Keywords:** preclinical, phase I, tyrosine kinase inhibitor, MET, AXL, VEGFR2

## Abstract

**Background:**

CT053PTSA is a novel tyrosine kinase inhibitor that targets MET, AXL, VEGFR2, FLT3 and MERTK. Here, we present preclinical data about CT053PTSA, and we conducted the first-in-human (FIH) study to evaluate the use of CT053PTSA in adult patients with pretreated advanced solid tumors.

**Methods:**

The selectivity and antitumor activity of CT053PTSA were assessed in cell lines *in vitro* through kinase and cellular screening panels and in cell line-derived tumor xenograft (CDX) and patient-derived xenograft (PDX) models *in vivo*. The FIH, phase I, single-center, single-arm, dose escalation (3 + 3 design) study was conducted, patients received at least one dose of CT053PTSA (15 mg QD, 30 mg QD, 60 mg QD, 100 mg QD, and 150 mg QD). The primary objectives were to assess safety and tolerability, to determine the maximum tolerated dose (MTD), dose-limiting toxicity (DLT), and the recommended dose of CT053PTSA for further study. Secondary objectives included pharmacokinetics, antitumor activity.

**Results:**

CT053 (free-base form of CT053PTSA) inhibited MET, AXL, VEGFR2, FLT3 and MERTK phosphorylation and suppressed tumor cell angiogenesis by blocking VEGF and HGF, respectively, *in vitro*. Moreover, cell lines with high MET expression exhibited strong sensitivity to CT053, and CT053 blocked the MET and AXL signaling pathways. In an *in vivo* study, CT053 significantly inhibited tumor growth in CDX and PDX models. Twenty eligible patients were enrolled in the FIH phase I trial. The most common treatment-related adverse events were transaminase elevation (65%), leukopenia (45%) and neutropenia (35%). DLTs occurred in 3 patients, 1/6 in the 100 mg group and 2/4 in the 150 mg group, so the MTD was set to 100 mg. CT053PTSA was rapidly absorbed after the oral administration of a single dose, and the C_max_ and AUC increased proportionally as the dose increased. A total of 17 patients in this trial underwent tumor imaging evaluation, and 29.4% had stable disease.

**Conclusions:**

CT053PTSA has potent antitumor and antiangiogenic activity in preclinical models. In this FIH phase I trial, CT053PTSA was well tolerated and had a satisfactory safety profile. Further trials evaluating the clinical activity of CT053PTSA are ongoing.

## 1 Introduction

Cellular signaling networks and interactions regulate the biological capabilities of cancer cells (1). One of the most important signaling pathways in the cell signal transduction network is the tyrosine kinase receptor pathway. Receptor tyrosine kinases (RTKs) are a subclass of tyrosine kinases that are involved in mediating cell-to-cell communication and controlling a wide range of complex biological functions, including cell growth, motility, differentiation, metabolism and immune responses (2, 3).Dysregulation of RTK signaling leads to many human diseases, especially cancer, making these proteins promising drug targets for cancer therapy (4). Cellular-mesenchymal to epithelial transition factor (c-MET, MET) is an RTK with one known ligand, hepatocyte growth factor (HGF), and it is expressed by endothelial cells, epithelial cells, hepatocytes, neurons, and hematopoietic cells (5). Aberrant activation of the MET/HGF pathway is involved in the development and metastatic progression of various tumors, and this pathway constitutes the mechanism by which cancer patients acquire resistance to targeted treatment and attenuates tumor response to immunotherapy (6–11). In addition, overexpression of MET is observed in many cancers and is a poor prognostic factor for survival (12–16). AXL is an RTK that belongs to the TAM (TYRO3, AXL, and MERTK) family, and activation of AXL has been implicated in promoting cell proliferation, migration, invasion, and epithelial-mesenchymal transition (EMT); mediating drug resistance; affecting the immune response; and indicating poor prognosis in several cancers (17–21). Aberrant expression of MERTK, another TAM RTK, also contributes to chemotherapy resistance, colony-forming potential, migration, EMT induction and involves in main process of anti-tumoral immunity in tumor cells (3, 22–25). Vascular endothelial growth factors (VEGFs) regulate vascular development, angiogenesis and lymphangiogenesis by binding to a number of receptors during the process of cancer angiogenesis. Recently, VEGFs are found immunosuppressive by inhibiting functional T cells, increasing the recruitment of suppressive immune cells, and hindering dendritic cells differentiation and activation (26). VEGFR-2 is the major RTK that regulates VEGF-induced vascular endothelial function (27). Moreover, MET, VEGFR-2 and AXL transactivation and synergistic interactions promote the activation of downstream signaling (28–30). RTKs that target MET, VEGFR-2 and AXL significantly increase the tumor suppressive effect and reduce the chance of tumor resistance to treatments.

CT053PTSA is a potent inhibitor of MET, AXL, VEGFR-2, FMS-like tyrosine kinase 3 (FLT3) and MERTK, all of which have been implicated in tumor pathogenesis. CT053PTSA (CT053 in a free-base form) was synthesized by HEC R&D Center, Sunshine Lake Pharma Co., Ltd. The nuclear structure and target of CT053PTSA are similar to those of cabozantinib, an antitumor drug marketed by Exelixis in the United States. The molecular formula of CT053PTSA is C31H29FN4O5 • C7H8O3S, and the molar mass is 728.29. The objectives of this study were to assess the antitumor activity of CT053PTSA *in vivo* and in MET-dependent tumor xenografts. Moreover, a FIH, phase I, single-center, single-arm, dose escalation (3 + 3 design) study was conducted in patients with advanced solid tumors who had previously received treatment. The primary objectives were to assess safety and tolerability as well as to determine the maximum tolerated dose (MTD), dose-limiting toxicity (DLT), and the dose of CT053PTSA recommended for further study. The secondary objectives included elucidating the pharmacokinetics and antitumor activity of CT053PTSA. The study is registered with ClinicalTrials.gov as NCT04577703.

## 2 Materials and methods

### 2.1 Compound and biochemical kinase binding assay

**Figure 1:**
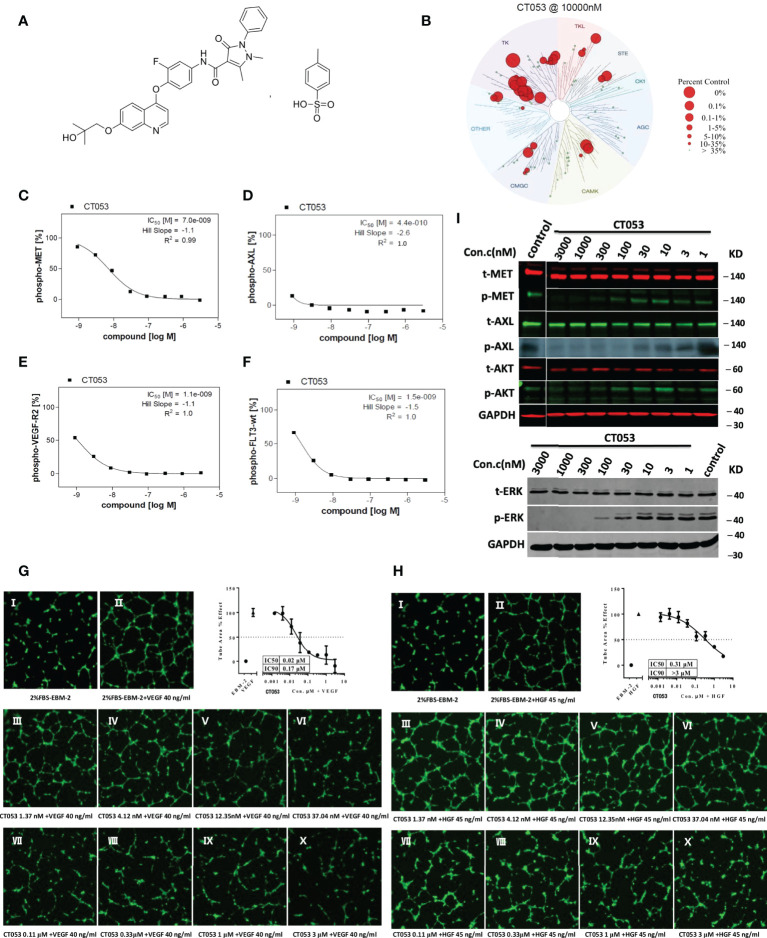
Chemical structure, cellular inhibitory effect and tubule formation effect of CT053PTSA *in vivo*. **(A)** Chemical structure of CT053PTSA. **(B)** Small molecule kinase interaction maps for CT053. CT053 was screened against a KinomeScan (http://www.kinomescan.com) panel of 95 kinase assays. Red circles indicate kinases bound, and circle size indicates binding affinity. Cellular inhibitory effect of CT053 on kinase phosphorylation: CT053 inhibited the phosphorylation of MET in MKN45 cells, and the IC50 value was 7.0 nM **(C)**. AXL was examined in AXL-MEFs cells, and the IC50 value was 0.44 nM **(D)**. VEGFR-2 and FLT3 were examined in HUVECs and FLT-wt-MEFs, and the IC50 values were 1.1 nM **(E)** and 1.5 nM **(F)**. CT053 inhibits VEGF-induced **(G)** and HGF-induced **(H)** tubule formation. Tubule formation was visualized in HUVECs incubated with 2% FBS-EBM-2 as a control and with VEGF at a concentration of 40 ng/ml and HGF at a concentration of 45 ng/ml. Elevated concentrations of CT053 (1.37 nm (III), 4.12 nm (IV), 12.35 nm (V), 37.04 nm (VI), 0.11 μM (VII), 0.33 μM (VIII), 1 μM (IX), and 3 μM (X)) were coincubated with VEGF/HGF, the percentage area of tube formation stimulated by VEGF/HGF decreased, and the IC50 values were 0.02 μM and 0.31 μM. **(I)** Inhibition of the phosphorylation of MET, AXL and signaling molecules (AKT and ERK) in SNU-5 cells. SNU-5 cells were treated with different dilutions of CT053 (3000 nm, 1000 nm, 300 nm, 100 nm, 30 nm, 10 nm, 3 nm, and 1 nm), and MET autophosphorylation, AXL phosphorylation, AKT phosphorylation, and ERK phosphorylation were all inhibited by CT053. AXL phosphorylation was completely suppressed by 100 nM CT053, and the phosphorylation of MET and its downstream protein ERK was inhibited by 1000 nM CT053. A higher concentration of CT053 was required to suppress the induced activity of AKT.

CT053PTSA (**Figure 1A**) was synthesized by HEC R&D Center, Sunshine Lake Pharma Co., Ltd. The results of biochemical kinase binding assays and kinase inhibition assays are shown in the Supplemental Materials.

### 2.2 Cellular kinase phosphorylation assay

Phosphorylation of the receptors MET, AXL, VEGFR2, and FLT3 was assessed in the transgenic human MKN45 gastric tumor cell line overexpressing MET, mouse embryonic fibroblasts (MEFs) expressing full-length human AXL (AXL-MEFs), human umbilical vein endothelial cells (HUVECs) overexpressing VEGFR-2, and MEFs expressing full-length human FLT3 (FLT3-wt-MEFs), respectively. The cells were serum starved overnight and then incubated with the compound CT053 diluted in gradient concentration in serum-free medium for 90 min at 37C. Then, HUVECs and FLT3-wt-MEFs were stimulated with the ligands VEGF-A (100 ng/ml, for 3 min) and FLT3-ligand (50 ng/ml, for 5 min). MKN45 (overexpressing MET) and AXL-MEFs cells had no stimulation. Cells treated with staurosporine (AXL, FLT3-wt, c-Kit, FLT3, TIE2, VEGF-R2; [1,0E-05 M]) or BMS777607 (MET; [1,0E-06 M]) or sunitinib (VEGF-R3; [1,0E-06 M]) or crizotinib (RON; [1,0E-05 M] were defined as low control (n = 8). The median value of those wells represented the background and was set to 0%. Cells treated with solvent alone were defined as high control (n = 8). The median value of those wells was set to 100%. Test sample-treated cells as well as high and low control were stimulated in identical manner. Quantification of receptor tyrosine kinase autophosphorylation was assessed in 96-well plates *via* sandwich ELISA using a receptor specific capture antibody and an anti-phosphotyrosine detection antibody. Raw data were converted into percent substrate phosphorylation relative to high controls, which were set to 100%. IC50 values were determined using GraphPad Prism 5 software with constrain of bottom to 0 and top to 100 using a nonlinear regression curve fit with variable hill slope.

### 2.3 Endothelial cell tubule formation assay

HUVECs were incubated with serial dilutions of the compound CT053 (1.37 nM, 4.12 nM, 12.35 nM, 37.04 nM, 0.11 μM, 0.33 μM, 1 μM, or 3 μM) for 8 hours in the presence or absence of VEGF (40 ng/ml, R&D, MN, USA) or HGF (45 ng/ml, R&D, MN, USA). Then, after staining with Calcein AM (ThermoFisher, CA, USA), images of tubule formation were captured, and the total tube area was quantified with ImageXpress HCS (Molecular Devices, CA, USA).

### 2.4 Cell lines and cell proliferation assay

Human gastric tumor cell lines with high MET expression levels, namely, the GTL-16, MKN45, SNU638, Hs746T, SNU-620, and SNU-5 cell lines, were used in the assay. These cell lines were obtained from American Type Culture Collection (ATCC), Japanese Collection of Research Bioresources (JCRB), Shanghai Institutes of Biological Sciences, CAS (SIBS), Korean Cell Line Bank (KCLB) or National Institutes of Health (NIH). The cell lines were cultured overnight in 96-well plates and then treated with serial dilutions of the compound CT053. Cell viability was measured after 72 hours using a CellTiter-Glo Luminescent Cell Viability Assay Kit (Promega, WI, USA) according to the manufacturer’s instructions.

### 2.5 mRNA expression

The MET mRNA expression level in each human gastric cell lines was evaluated with real-time PCR. Glyceraldehyde-3-phosphate dehydrogenase (GAPDH) was considered as the internal control. The amplification was performed in a 20 μl mixture containing 10 μl of TaqMan Universal PCR Master Mix (Invitrogen, CA, USA) with UNG, 1 μl of the MET (Hs01565584_m1) or GAPDH (Hs02786624_g1) TaqMan Gene Expression Assay (Thermo, CA, USA), and 5 μl of cDNA solution. Each sample was analyzed in triplicate on an Agilent Technologies Stratagenes Mx3005P (CA, USA). Relative MET mRNA expression is presented as the fold-change, which was calculated using the 2^ (-ΔΔCt) method.

### 2.6 Western blot assay

The SNU-5 cell line was selected for western blotting analysis of signaling molecules. After CT053 treatment, proteins were extracted by protein lysis, and the process of western blotting analysis included adding the samples to SDS-PAGE gels, transferring the proteins to polyvinylidene fluoride membranes (Millipore, MA, USA), blocking the membranes with 5% nonfat milk in TBST, incubating the membranes with the primary antibody, probing the membranes with a peroxidase-linked secondary antibody, and visualizing the protein bands. Immunoreactive proteins (p-AXL, t-ERK and p-ERK) were visualized using the ECL system (Pierce Chemical, TX, USA). Other proteins were detected by Odyssey (LI-COR, NE, USA) at a wavelength of 700 nm or 800 nm. In addition, we also examined the effects of CT053 on the phosphorylation of MET in the GA3121 PDX model by western blotting. Tumors were harvested after 21 days of treatment with vehicle or CT053. Tumor lysates were subjected to western blotting. The following antibodies were purchased from Cell Signaling Technology (Cell Signaling Technology Cat#3148, RRID : AB_1031042): anti-MET (Cell Signaling Technology Cat# 3148, RRID : AB_1031042), anti-p-MET Y1234/1235 (Antibodies-Online Cat# ABIN461461, RRID : AB_10786128), anti-AXL (Cell Signaling Technology Cat# 4566, RRID : AB_2062563), anti-p-AXL Y702 (5724S), anti-ERK (9107S), anti-p-ERK Thr202/Tyr204 (4370S), anti-p-AKT Ser473 (4060S), and anti-AKT (Cell Signaling Technology Cat# 2920, RRID : AB_1147620).

### 2.7 *In vivo* efficacy study

Human tumor xenografts were established in female BALB/cA nude mice (5-6 weeks old) by administering the SNU-5 and MKN-45 cells, which have high MET expression levels. For efficacy studies in patient-derived tumor xenograft (PDX) mouse models, MET-overexpressing GA0046 and GA3121 tumors (obtained from Crownbio, CA, USA) were established by directly implanting surgical tissues into immunodeficient mice. Tumor-bearing mice were randomized into groups (8 animals per group) according to their tumor sizes, and when the average tumor volume reached approximately 150 mm^3^, the mice were treated with vehicle or CT053 at doses of 1, 3, 10 or 20 mg/kg once per day by oral gavage for 16 or 21 days. The subcutaneous tumor size and body weight were measured twice weekly. The tumor volumes were determined by measuring two perpendicular diameters with calipers and calculated as (length × width^2^)/2. Tumor growth inhibition (TGI: TGI% = (1 - tumor volume (treatment)/tumor volume (control) ×100) was calculated to evaluate the effect of the treatment on tumor growth inhibition. Tumors were harvested from the GA3121 PDX model mice after 21 days of treatment with vehicle or CT053 at a dose of 20 mg/kg to perform further experiment.

### 2.8 Clinical study

#### 2.8.1 Study design

This was a single-center, single-arm, dose-escalation phase I study in patients with advanced solid tumors who failed to achieve an effect with or who had not received standard systemic anticancer treatment. Eligible patients had at least one measurable lesion per RECIST v 1.1, Eastern Cooperative Oncology Group (ECOG) performance status of 0 or 1, and adequate hematological, renal, cardiac and hepatic functions. The primary objectives were to assess safety and tolerability as well as to determine the MTD, DLT, and dose recommended for further study. The secondary objectives were to assess the PK and preliminary efficacy of CT053PTSA.

CT053PTSA was administered orally, and this dose-escalation trial included 5 dose levels: 15 mg QD, 30 mg QD, 60 mg QD, 100 mg QD, and 150 mg QD. An initial accelerated titration design was used in the 15 and 30 mg QD cohorts, and the “3+3” schema was used starting in the 60 mg QD cohort. DLT was defined as any of the following events occurring during cycle 0 (7-day wash-out period after a single dose) and cycle 1 (multiple doses, including a 7-day observation period after 28 days of administration) per Common Terminology Criteria for Adverse Events (CTCAE) version 4.0: G4 neutropenia lasting ≥ 7 days; febrile neutropenia with fever ≥ 38.5°C; G3 thrombocytopenia associated with bleeding or G4 thrombocytopenia lasting ≥ 4 days; grade ≥ 3 nausea/vomiting/diarrhea/hypertension not alleviated after 4 days of supportive care; any other grade ≥ 3 nonhematological toxicities (excluding alopecia, hyperuricemia without gout). The study protocol was approved by an institutional review board at Sun Yat-sen University Cancer Center. Written informed consent was obtained from all the patients before enrollment.

#### 2.8.2 Patient treatment and pharmacokinetic analysis

A clinical pharmacokinetic study of CT053PTSA was performed for both single doses and multiple doses. Blood samples were collected from each subject at prespecified times before and after single-dose administration (systemic blood samples: before treatment and 0.5, 1, 2, 3, 4, 5, 8, 12, 24, 36, 48, 72, 120 and 168 hours after treatment) and during multiple dosing (cycle 1 before treatment on Days 8, 15 and 22, as well as systemic blood samples on Day 28).

#### 2.8.3 Assessments

Descriptive statistics of safety are presented per CTCAE v4.0. Subjects who received at least one dose of CT053PTSA were considered eligible to be evaluated and included in safety analyses. Single-dose and multiple-dose PK parameters included peak concentration (C_max_); time to reach C_max_ (T_max_); the area under the plasma concentration time curve from 0 to 168 hours, at 24 hours, and infinity (AUC_0-168_, AUC_0-24_, AUC_0-inf_); and oral clearance (CL/F) and apparent volume of distribution (Vz/F) for both CT053PTSA and its metabolites: CT053-M1. According to the RECIST 1.1 criteria, imaging examinations were performed at each specified time point in the protocol. The antitumor activity was measured by RECIST1.1.

### 2.9 Statistical analysis

Statistical analysis of CT053-treated tumors versus vehicle-treated tumors or versus nontreated tumors was performed by repeated-measures ANOVA. The data from the tumor xenograft models were expressed as the means ± standard error of the mean (SEM) and were plotted as a function of time. Noncompartmental pharmacokinetic analysis was performed using Phoenix WinNonlin Version 6.2.1 (Certara Inc., NJ, USA). Demographics and laboratory results of all the subjects were summarized using descriptive statistics. The pharmacokinetic parameters T_max_ and T_1/2_ were shown as the median (min, max) and mean ± SD, respectively. A two-sided *P* < 0.05 was considered to indicate statistical significance. Statistical analysis was performed by using GraphPad Prism 5.0 (GraphPad Prism, RRID : SCR_002798) and SPSS software version 19.0 (SPSS, RRID : SCR_002865).

## 3 Results

### 3.1 Kinase selectivity and activity

To assess the potential off-target inhibitory activity of CT053 on other kinases, we screened the compounds against a KinomeScan panel of 95 kinase binding assays. The highest affinity target identified for CT053 was the tyrosine kinase family (**Figure 1B**), which indicated that the kinase interaction pattern for CT053 was highly specific.

A follow-up IC50 calculation was performed with 8 concentrations of CT053 against 27 kinases selected from the first screening step. CT053 is a potent inhibitor of AXL, with an IC50 value of 3.4 nM. CT053 strongly inhibited several kinases, including MET, VEGFR2, MERTK and FLT3 (IC50 = 68, 46, 14, 8.6 nM, respectively) (**Supplementary Table 1**). In cellular assays, CT053 inhibited the phosphorylation of MET, AXL, VEGFR2, and FLT3 with IC50 values of 7.0, 0.44, 1.1, and 1.5 nM, respectively (**Figures 1C-F**).

### 3.2 CT053 inhibits endothelial cell tubule formation *in vitro*

We further investigated the effect of CT053 on angiogenesis *in vitro*. The results showed that as the concentration of CT053 increased (1.37 nM, 4.12 nM, 12.35 nM, 37.04 nM, 0.11 μM, 0.33 μM, 1 μM, or 3 μM), the percentage area of tube formation stimulated by VEGF and HGF decreased, and the IC50 values were 0.02 and 0.31 μM, respectively (**Figures 1G, H**). These data suggest that CT053 inhibits tumor cell angiogenesis *in vitro*.

### 3.3 Potent anti-proliferation effects of CT053 depend on MET expression

CT053 potently inhibits the tyrosine kinase activity of recombinant MET and the phosphorylation of MET in *in vitro* cell-based assays, with IC50 values of 68 nM and 7.0 nM, respectively (**Supplementary Table 1** and **Figure 1C**). To investigate biomarkers that could help identify tumors that are sensitive to MET kinase inhibition, an *in vitro* antiproliferative screen was performed using a gastric tumor cell line panel. Six cell lines (SNU-5, SNU638, SNU-620, GTL-16, MKN45, and Hs746T cells) showed high MET mRNA levels with a median ratio value of 18.75 (range: 5.64-28.80), whereas the other eleven cell lines showed significantly lower MET mRNA levels with a median ratio value of 1.01 (range: 0.01-3.27) (*P* < 0.001) (**Supplementary Table 2**). Cell lines with high MET mRNA levels exhibited strong sensitivity to CT053, with an average IC50 value of 0.095 μM. However, CT053 had a very weak inhibitory effect on cell lines with low MET mRNA levels, with an average IC50 value of more than 10 μM (*P* < 0.001) (**Supplementary Table 2**). Previous studies reported that MET gene expression has been linked to the sensitivity of cell lines to MET inhibitors (31, 32). Our results demonstrate that sensitivity to CT053PTSA is associated with MET transcription and mRNA expression *in vitro*.

### 3.4 CT053 blocks MET, AXL and downstream signaling pathways

To show the effect of CT053 on the MET and AXL signaling pathways, SNU-5 cells were treated with serial dilutions of CT053. The results showed that CT053 inhibited MET autophosphorylation. Moreover, at 100 nM, CT053 completely suppressed AXL phosphorylation, and at 1000 nM, CT053 completely suppressed the phosphorylation of MET and its downstream protein ERK. However, a much higher concentration of CT053 was required to suppress the induced AKT activity (**Figure 1I**).

### 3.5 CT053 inhibits tumor growth in a MET-dependent manner in tumor cell line-derived and patient-derived tumor xenograft models

**Figure 2:**
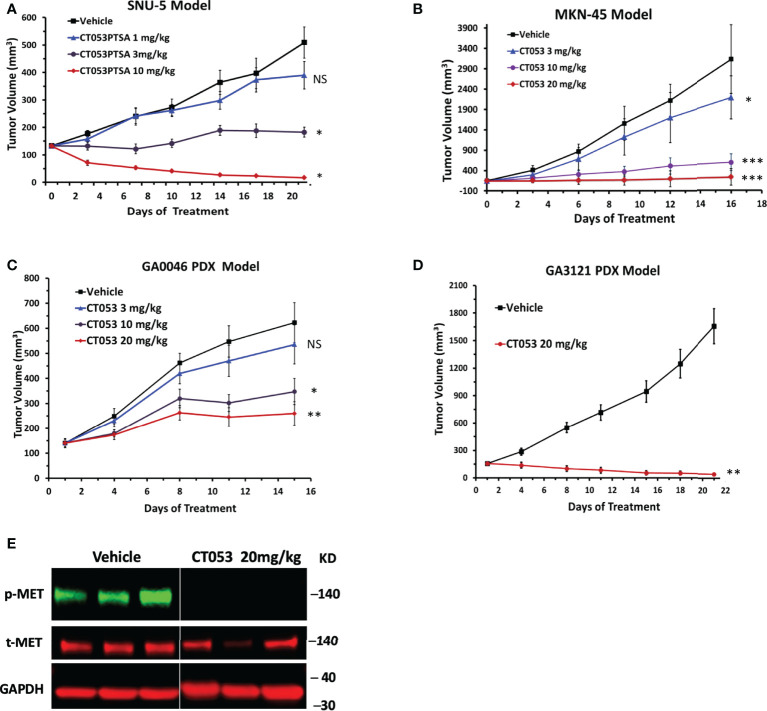
CT053 inhibits tumor growth in a MET-dependent manner in tumor xenograft models. Mice bearing SNU-5 CDX xenografts **(A)**, MKN45 CDX xenografts **(B)**, GA0046 PDX xenografts **(C)** or GA3121 PDX xenografts **(D)** were treated orally once daily with vehicle or CT053 at doses of 1, 3, 10 or 20 mg/kg. The tumor volume was measured twice per week, and the data represent (n = 8) the mean ± standard error of the mean (SEM) for each group. An adjusted value of *P* < 0.05 was considered statistically significant. Each treatment group was compared to the control group (*, *P* < 0.05; **, *P* < 0.01; ***, *P* < 0.001; NS = not significant). Statistical significance was determined by repeated-measures ANOVA. **(E)** Effects of CT053 treatment on the phosphorylation of MET in the PDX model. Samples were harvested from the GA3121 PDX model mice after 21 days of treatment with vehicle or CT053 at a dose of 20 mg/kg.

We performed two *in vivo* efficacy studies in cell line-derived tumor xenograft (CDX) models (derived from the SNU-5 and MKN45 cell lines) and two *in vivo* antitumor efficacy studies in PDX models (GA0046 and GA3121). CT053 significantly inhibited tumor growth compared with vehicle, exhibiting TGI values of 32% (*P* = 0.88), 87% (*P* = 0.018) and 187% (*P* = 0.017) at doses of 1, 3, and 10 mg/kg in the SNU-5 CDX model (**Figure 2A**) and TGI values of 31% (*P* = 0.014), 85% (*P* < 0.001), 97% (*P* < 0.001) at doses of 3, 10 and 20 mg/kg in the MKN-45 CDX model, respectively (**Figure 2B**). Furthermore, CT053 also had a potent inhibitory effect on tumor growth, with TGI values of 18% (*P* = 0.88), 57% (*P* = 0.01), and 75% (*P* = 0.002) at doses of 3, 10, and 20 mg/kg in the GA0046 PDX model, respectively (**Figure 2C**), and a TGI value of 172% (*P* = 0.001) at 20 mg/kg CT053 in the GA3121 model (**Figure 2D**). In addition, we found that the phosphorylation of MET was significantly inhibited in the CT053-treated group compared with the vehicle group in the GA3121 PDX model (**Figure 2E**). These results indicated that CT053 suppressed tumor growth in the CDX and PDX models with high MET expression in a dose-dependent manner, and the attenuation of tumor growth by CT053 most likely occurs due to its inhibition of MET signaling activity. Body weight was measured to monitor the toxicity and there was no significant change in body weight for all groups.

### 3.6 Clinical study

#### 3.6.1 Patients

**Figure 3:**
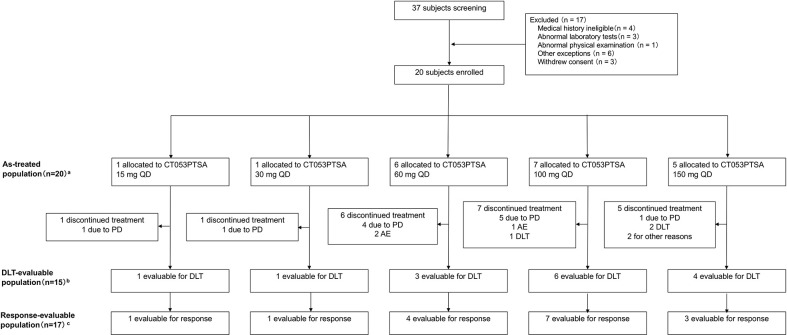
Flow chart of patients enrolled in the study. A total of 20 patients received at least one dose of CT053PTSA. ^a^The as-treated population included patients who received any dose of CT053PTSA, grouped according to actual dose received; ^b^The population that was eligible to for DLT assessment included all the patients enrolled in the dose-escalation phase who received one full dose of CT053PTSA and completed the safety follow-up through the DLT evaluation period, defined as the time during cycle 0 (7 days after a single dose) and cycle 1 (multiple doses, including a 7-day observation period after 28 days of administration), or who experienced any DLT during this period; ^c^The population that was eligible for response assessment included all patients in the as-treated population who had ≥ 1 postbaseline tumor assessment or alternatively who died from any cause or who discontinued treatment due to clinical PD prior to any postbaseline tumor assessment. AE, adverse event; PD, progressive disease; DLT, dose-limiting toxicity.

A total of 20 patients were enrolled in the study (**Figure 3**). The median age was 47.3 years (range: 28-66 years), and all of the patients received at least one dose of CT053PTSA and were included in the safety analysis set from February 2014 to December 2015. The baseline characteristics are summarized in **Table 1**.

**Table 1:** Baseline patient demographics and clinical characteristics.

Characteristics	All patients (N = 20)
**Age, median (range)**	47.3 (28-66)
**Gender, n (%)**	
** Male**	16 (80)
** Female**	4 (20)
**ECOG, n (%)**	
** 0**	14 (70)
** 1**	6 (30)
**Tumor type, n (%)**	
** Nasopharyngeal carcinoma**	15 (75)
** Lung adenocarcinoma**	2 (10)
** Lip and/or oral cavity cancer**	1 (5)
** Pleural mesothelioma**	1 (5)
** Rectal cancer**	1 (5)
**Number of metastasis organs, n (%)**	
** 1**	3 (15)
** 2**	8 (40)
** ≥ 3**	9 (45)
**Metastasis site, n (%)**	
** Liver**	10 (50)
** Lung**	13 (65)
** Others**	10 (50)
**Number of prior systemic regimens, n (%)**	
** < 2**	5 (25)
** ≥ 2**	15 (75)

ECOG, Eastern Cooperative Oncology Group.

#### 3.6.2 Safety, tolerability and recommended dose of CT053PTSA in patients

Nineteen (95%) patients experienced at least one adverse event (AE), and of these patients, treatment-related adverse events (TRAEs) were reported in 16 (80%). The most frequently reported (≥ 20%) TRAEs of any grade included transaminase elevation (65%), leukopenia (45%), neutropenia (35%), decreased platelet count (30%), decreased appetite (30%), and hypertension (20%) (**Table 2**). Most AEs were grade 1-2 and easily managed. Only 3 patients (15%, 100 mg QD: n = 1; 150 mg QD: n = 2) experienced grade 3 TRAEs, including decreased platelet count (10%), elevated transaminase (5%), leukopenia (5%), decreased blood sodium (5%), decreased appetite (5%), hypertension (5%) and stomatitis (5%), during treatment. One serious adverse event (SAE) was reported at 150 mg, which was grade 3 stomatitis (also a DLT) leading to hospitalization. Regarding DLTs, one out of six patients who were eligible for DLT assessment experienced a DLT (grade 3 transaminase elevation) at 100 mg QD. Two of four patients who were eligible for DLT assessment at 150 mg QD experienced DLTs, namely, grade 3 blood sodium decreased, grade 3 decreased appetite, grade 3 hypertension and grade 3 stomatitis. Thus, the MTD was 100 mg, and the recommended dose for further study of CT053PTSA was determined to be 60~100 mg QD.

**Table 2:** Treatment-related adverse events reported in all patients.

Event, n (%)	15mg (n = 1)		30mg (n = 1)		60mg (n = 6)		100mg (n = 7)		150mg (n = 5)		All (n = 20)	
	All	≥ G3	All	≥ G3	All	≥ G3	All	≥ G3	All	≥ G3	All	≥ G3
**Investigations**												
**transaminase elevation**	1 (100)	0	0	0	3 (50)	0	6 (85.7)	1 (14.3)	4 (80)	0	13 (65)	1 (5)
**leukopenia**	1 (100)	0	0	0	1 (16.7)	0	4 (57.1)	0	3 (60)	1 (20)	9 (45)	1 (5)
**neutropenia**	0	0	0	0	1 (16.7)	0	4 (57.1)	0	2 (40)	0	7 (35)	0
**Platelet count decreased**	0	0	0	0	1 (16.7)	0	4 (57.1)	1 (14.3)	1 (20)	1 (20)	6 (30)	2 (10)
**Electrocardiogram QT prolonged**	0	0	0	0	1 (16.7)	0	1 (14.3)	0	1 (20)	0	3 (15)	0
**Hemoglobin decreased**	0	0	0	0	0	0	0	0	1 (20)	0	1 (5)	0
**Blood creatinine increased**	0	0	0	0	0	0	0	0	1 (20)	0	1 (5)	0
**Blood potassium decreased**	0	0	0	0	0	0	0	0	1 (20)	0	1 (5)	0
**Blood chloride decreased**	0	0	0	0	0	0	1 (14.3)	0	0	0	1 (5)	0
**Blood sodium decreased**	0	0	0	0	0	0	0	0	1 (20)	1 (20)	1 (5)	1 (5)
**Blood uric acid increased**	0	0	0	0	0	0	0	0	1 (20)	0	1 (5)	0
**Metabolism and nutrition disorders**												
**Decreased appetite**	0	0	1 (100)	0	0	0	2 (28.6)	0	3 (60)	1 (20)	6 (30)	1 (5)
**Hyponatremia**	0	0	1 (100)	0	0	0	1 (14.3)	0	0	0	2 (10)	0
**Electrolyte imbalance**	0	0	0	0	0	0	0	0	1 (20)	0	1 (5)	0
**Vascular disorders**												
**Hypertension**	0	0	0	0	0	0	2 (28.6)	0	2 (40)	1 (20)	4 (20)	1 (5)
**Gastrointestinal disorders**												
**Nausea**	0	0	0	0	0	0	1 (14.3)	0	1 (20)	0	2 (10)	0
**Diarrhea**	0	0	0	0	0	0	1 (14.3)	0	1 (20)	0	2 (10)	0
**Stomatitis**	0	0	0	0	0	0	1 (14.3)	0	1 (20)	1 (20)	2 (10)	1 (5)
**Dry mouth**	0	0	0	0	0	0	1 (14.3)	0	0	0	1 (5)	0
**Mouth ulceration**	0	0	0	0	0	0	0	0	1 (20)	0	1 (5)	0
**Vomiting**	0	0	0	0	0	0	0	0	1 (20)	0	1 (5)	0
**Skin and subcutaneous tissue disorders**												
**Palmar-plantar erythrodysaesthesia syndrome**	0	0	0	0	0	0	1 (14.3)	0	0	0	1 (5)	0
**General disorders and administration site conditions**												
**Pyrexia**	0	0	0	0	0	0	0	0	1 (20)	0	1 (5)	0
**Pain**	0	0	0	0	0	0	1 (14.3)	0	0	0	1 (5)	0
**Nervous system disorders**												
**Dizziness**	0	0	0	0	0	0	1 (14.3)	0	0	0	1 (5)	0
**Respiratory, thoracic and mediastinal disorders**												
**Oropharyngeal pain**	0	0	0	0	0	0	1 (14.3)	0	0	0	1 (5)	0
**Endocrine disorders**												
**Hypoparathyroidism**	0	0	0	0	0	0	0	0	1 (20)	0	1 (5)	0
**Renal and urinary disorders**												
**Proteinuria**	0	0	0	0	0	0	1 (14.3)	0	0	0	1 (5)	0
**Cardiac disorders**												
**Sinus bradycardia**	0	0	0	0	0	0	0	0	1 (20)	0	1 (5)	0
**Blood and lymphatic system disorders**												
**Anaemia**	0	0	0	0	0	0	1 (14.3)	0	0	0	1 (5)	0

All adverse events were characterized and graded per Common Terminology Criteria for Adverse Events v4.0.

#### 3.6.3 Pharmacokinetics of CT053PTSA in patients

**Figure 4:**
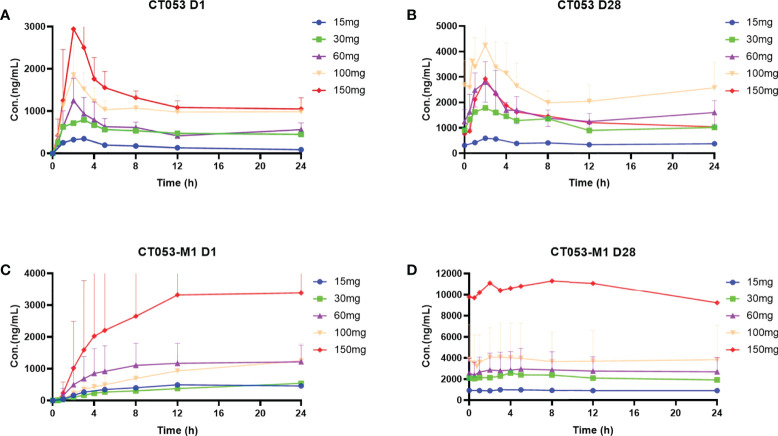
Concentration-time profiles (mean ± SD) of plasma CT053PTSA and CT053-M1 levels by cohort after a single dose (Day 1) after multiple oral doses (Day 28). **(A)** CT053PTSA levels after a single dose. **(B)** CT053PTSA levels after multiple oral doses. **(C)** CT053-M1 levels after a single dose. **(D)** CT053-M1 levels after multiple oral doses.

The PK parameters of CT053PTSA and its active metabolite CT053-M1 were evaluated after single- and multiple-dose administration. Prior to initiating a continuous daily treatment cycle, patients participated in a single-dose PK treatment period with a 7-day washout. Concentration-time plots of the plasma CT053PTSA and CT053-M1 levels in each cohort after a single dose (Day 1) or after multiple oral doses (Day 28) are shown in **Figure 4**, and the pharmacokinetic parameter summary is provided in **Supplementary Table 3**. CT053PTSA was rapidly absorbed after oral administration with a median T_max_ of 2~3 h for a single dose and 2 h for multiple doses. The observed mean half-life ranged from 24.7 to 33.9 hours for a single oral dose and from 13.8 to 38.4 hours for multiple oral doses. A steady state was reached by Day 8 following daily dosing. One or multiple peaks were observed after the C_max_ peak in the plasma concentration-time profiles within 48 h in most subjects. After a single oral dose, CT053 followed a general linear PK profile for doses from 15 to 150 mg, and a linear PK profile was also observed for doses from 60-100 mg after multiple doses. After oral administration, CT053-M1 was the most abundant metabolite in the plasma.

#### 3.6.4 Antitumor activities

We also evaluated the preliminary efficacy of CT053PTSA. A total of 17 patients underwent tumor imaging evaluation after taking CT053PTSA, and none of them achieved CR or PR. Five patients (29.4%) achieved SD. The DCR was 29.4%. Among these patients, two who received 60 mg QD CT053PTSA achieved SD for 16 weeks and 24 weeks. One patient in the 100 mg dose group achieved SD for 8 weeks.

**Figure 5:**
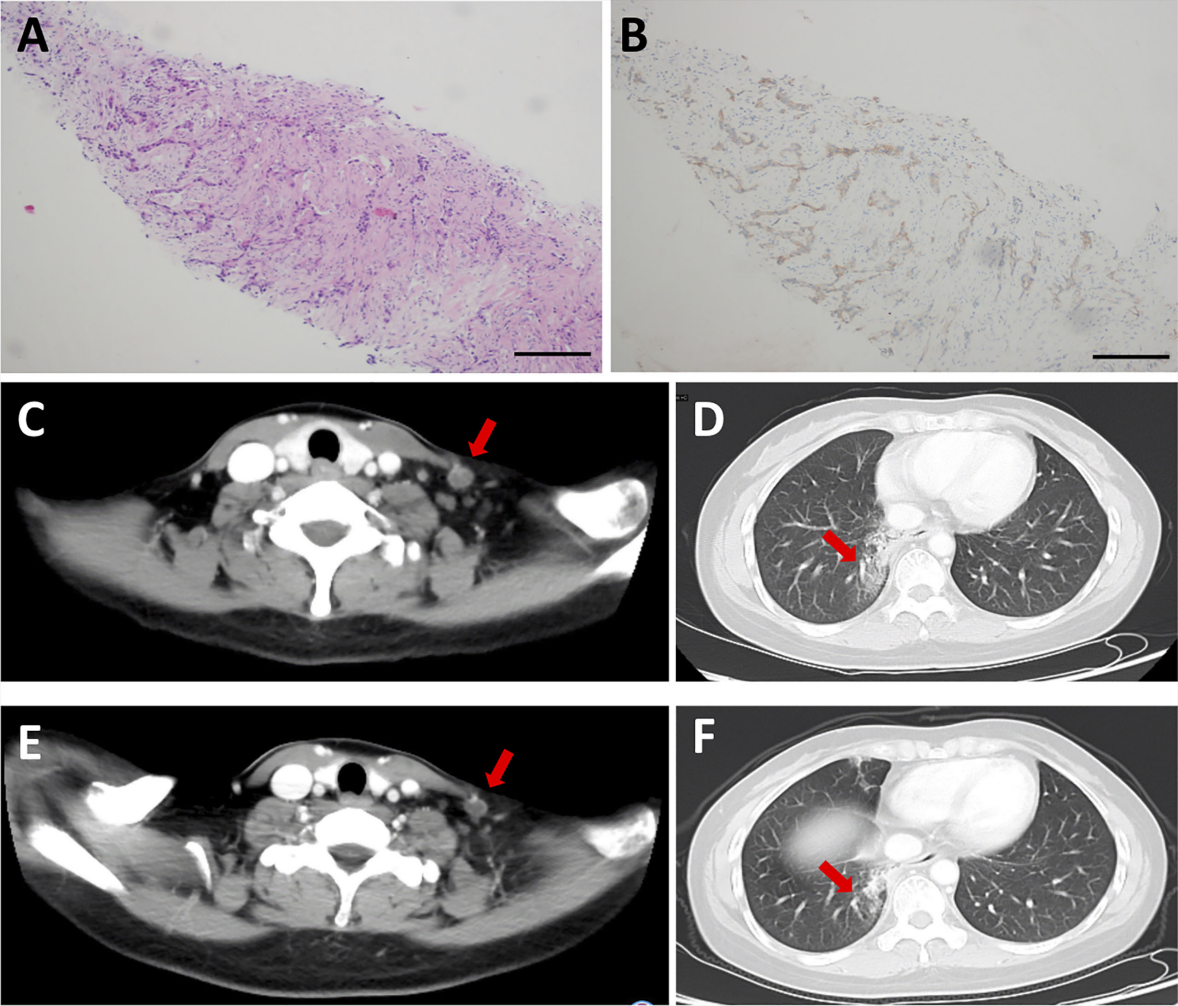
Representative H&E- and IHC-stained biopsy sections and CT images from a patient in the 60 mg CT053PTSA treatment group with advanced non-small cell lung cancer (EGFR, ALK negative) who achieved a durable stable response. **(A)** Representative H&E image of a stained biopsy from the left supraclavicular lymph node at baseline. **(B)** Representative IHC image of MET overexpression in a stained biopsy from the left supraclavicular lymph node at baseline. Baseline CT images (2014-11-24): **(C)** target lesion: left supraclavicular lymph node (red arrow) on enhanced CT. and **(D)**, nontarget lesion: lung metastases on enhanced CT. **(E, F)**, Cycle 2 Day 28 CT image (2015-02-04): target lesion and nontarget lesion at stable response.

Moreover, we analyzed the patient who achieved SD for 24 weeks. The patient was a 47-year-old female with EGFR exon 19-21 wild type, ALK negative non-small cell lung cancer (NSCLC). She had failed 1 line of platinum-based chemotherapy with a PFS of 27 months. She underwent a biopsy on neck lymph node before CT053PTSA treatment (**Figure 5A**), results showed that tumor tissue was MET overexpressed by immunohistochemistry (IHC) (**Figure 5B**). Baseline CT imaging showed a left supraclavicular lymph node (target lesion, **Figure 5C**) and multiple lung metastases (all < 1 cm, nontarget lesions, **Figure 5D**). The plasma of the patient was prospectively collection on the first day of cycle 1, 3, 6, whole exon sequencing of 416 genes was performed to assess circulating tumor DNA (ctDNA) with Illumina platform. As showed in **Supplementary Table 4**, targeted genes of CT053PTSA including AXL A79T mutation, FLT3 P893L mutation were eliminated compared baseline after CT053PTSA treatment. Other truncating mutations of tumor related genes (APC, BRCA1, EGFR, FLT4, etc) were also decreased rapidly during treatment. The patient received 6-time tumor assessments, the response was classified as SD (**Figure 5E**), and the nontarget lesions remained unchanged (**Figure 5F**). However, the patient experienced tumor progression after 6 cycles of CT053PTSA administration, and which point, the target lesion enlarged.

## 4 Discussion

CT053PTSA is a novel multikinase inhibitor with activity against MET, AXL, VEGFR2, FLT3 and MERTK, among others. In our study, CT053PTSA demonstrated potent inhibition of the phosphorylation of these kinases and tubule formation of tumor cells. Moreover, CT053PTSA showed significant antitumor efficacy in all models in the short term *in vivo*. Then, we conducted a FIH, phase I, single-center, single-arm, dose escalation clinical study, and the results indicated that CT053PTSA was well tolerated and safe from 15 mg QD to 100 mg QD. In addition, 100 mg QD was determined to be the MTD, and the recommended dose for further study of CT053PTSA was determined to be 60~100 mg QD.

Small molecule tyrosine kinase inhibitors (TKIs) that target tyrosine kinase receptor pathways have shown remarkable results in terms of specific therapeutic effects on various cancers. Inhibitors that target MET, AXL, VEGFR-2, FLT3 and MERTK are becoming research and development hotspots owing to their effect on promoting tumor development and progression. Cabozantinib is a multitarget RTK inhibitor that inhibits several RTKs, including MET, RET, VEGFR-1-3, KIT, FLT-3, TIE-2 (TEK tyrosine kinase, endothelial), tropomyosin-related kinase B (TRKB) and AXL, and it has been clinically approved for the treatment of medullary thyroid cancer (MTC) (33), renal cell cancer (RCC) (34), and hepatocellular carcinoma (HCC) (35).

Although CT053PTSA has a higher IC50 for MET and VEGFR-2 than cabozantinib, in cellular assays, CT053 inhibited the phosphorylation of MET, AXL, VEGFR2, and FLT3 with IC50 values of 7.0, 0.44, 1.1, and 1.5 nM, respectively, which were much lower than those of cabozantinib, with IC50 values of 7.8, 42, 1.9, and 7.5 mmol/L, respectively (36). Moreover, CT053PTSA inhibits the proliferation of HUVECs stimulated by HGF and VEGF to an extent that is comparable to cabozantinib. Crizotinib is a small molecule TKI that targets anaplastic lymphoma kinase (ALK), ROS1 and MET (37, 38). In cellular assays, crizotinib inhibited the phosphorylation of MET in NCI-H69 and HOP92 cells with IC50 values of 13 nM and 16 nM, respectively, which are higher than the values of CT053PTSA. Furthermore, the inhibition of anti-proliferation sensitivity and endothelial cell tubule formation by CT053PTSA *in vitro* was consistent with crizotinib (37). The results of *in vivo and in vitro* studies of CT053PTSA are comparable to those of cabozantinib and crizotinib, indicating that CT053PTSA could be a potent promising agent for treating tumors.

In the current FIH phase I study, the most common TRAEs were also commonly reported in studies of other MET inhibitors (39, 40). A phase I study of cabozantinib in Japanese patients with NSCLC demonstrated that 70% (16/23) of patients experienced ≥ grade 3 AEs, and the most common AEs included palmar-plantar erythrodysesthesia (100%), increased alanine aminotransferase (95%), increased aspartate aminotransferase (95%), hypertension (87%), and diarrhea (78%) (41). Merestinib is a multikinase inhibitor that targets MET, AXL and FLT3. A phase I study of merestinib showed that 40% (20/50) of patients experienced ≥ grade 3 TRAEs (42). Even though these were also observed in patients treated with CT053PTSA, they were much less frequently reported in our study, in which 15% of patients experienced ≥ grade 3 AEs. Twenty percent of patients experience hypertension, which is considered to be associated with the inhibition of VEGF-mediated angiogenesis (43). Hematologic toxicity included leukopenia (45%), neutropenia (35%), and decreased platelet count (30%), which are believed to be related to the inhibition of FLT3 (44). The TRAEs reported in this study were predominantly grade 1 and 2, and they were managed with supportive care. Three patients experienced DLTs, 2 patients treated with 150 mg QD, and one patient treated with 100 mg QD. CT053PTSA had a better safety profile, with no unexpected adverse reactions reported in this study. Therefore, according to the results of the study, the MTD was 150 mg, which was confirmed as the recommended dose for further study. Pharmacokinetic analysis indicated that CT053PTSA was rapidly absorbed after oral administration to patients with advanced solid tumors, with a median T_max_ of 2~3 h; this value was 4 h for cabozantinib (45). Exposure (C_max_ and AUC_0-24 h_) to CT053PTSA increased with dose. The PK data supported the QD regimen, and the half-life was reported to be 24.7-33.9 h, while it was 108-126 h for cabozantinib (45). Therefore, the QD regimen for CT053PTSA was determined for further development. We further investigated the preliminary efficacy of CT053PTSA. No CR or PR was observed, and 5 out of 17 patients achieved SD. The efficacy was acceptable because the patients who were enrolled all had heavily treated advanced solid tumors, accounted for a small number of patients, and were not selected based on MET, AXL, VEGFR-2, and FLT3 expression. We retrospectively found that the patient with MET overexpression had durable stable disease for approximately six months. In this case, the overexpression of MET was confirmed by IHC, and in our preclinical study, *in vivo* and *in vitro* studies were also performed on cells and models that overexpressed MET. The results indicated the consistency of the antitumor efficacy of CT053PTSA preclinical and clinical settings. Currently, ctDNA has been used for detecting early cancer occurrence (46) and predicting clinical response after treatment (47, 48). In the case report, after CT053PTSA administration, the amount of ctDNA gradually decreased and was undetectable at cycle 6. Furthermore, the abundance of truncation mutations in tumor suppressor genes was decreased after cycle 0 administration of CT053PTSA. Therefore, it is expected that CT053PTSA will have exhibit performance in cancer patients with MET, AXL, VEGFR-2 and FLT3 overexpression.

Epidermal growth factor receptor tyrosine kinase inhibitors (EGFR-TKIs) provide a favorable treatment outcome in EGFR mutation-positive NSCLC patients. However, their prognosis remains unfavorable because of the occurrence of either intrinsic or acquired resistance (49). Activation of alternate or bypass pathways occurs in 5-22% of patients with high MET expression (50, 51) and in 20% of patients with upregulated AXL expression (52), and these events account for acquired resistance to EGFR TKIs *via* the activation of downstream effectors, including NF-kappaB and AKT, or the promotion of EMT (53, 54). Our results demonstrated that CT053PTSA inhibited the phosphorylation of MET and AXL and blocked the MET, AXL and downstream signaling pathways. CT053PTSA combined with an EGFR-TKI will be a better treatment strategy for NSCLC. Based on the preclinical data and the results of this phase I study, subsequent clinical trials are ongoing and registered with ClinicalTrials.gov (NCT03758287).

Zhang Y et al. (11) found that lung cancer patients with MET amplification were resistant to immune checkpoint inhibitor and had a poor survival by down-regulating STING levels and antitumor T-cell infiltration. In addition, the study conducted by Li Q et al. (55) found that low-dose VEGFR2 blockade led to more infiltration and activation of immune cell, promoted generation of TGF-β, that in turn upregulated PD-1 expression on immune cells. Moreover, TAM (TYRO3, AXL, and MERTK) family also had negative influence on cancer immunotherapy by mediating efferocytosis, negative regulating of dendritic cell activity, and dysregulating production of chemokines (3). The studies indicated that targeting MET, AXL, and VEGFR2 may enhance the efficacy of immune checkpoint inhibitor for cancer patients, which can be explored in further study.

Our study also has several limitations. First, in the preclinical study, the tumor cell lines we used had high MET mRNA and/or protein overexpression levels, and we did not investigate the effect of CT053PTSA on models with MET mutations. Second, the phase I trial was performed in a single center, and the participants were all of Chinese descent, which may reduce the generalizability of the findings. Third, we did not evaluate the effect of food intake on the pharmacokinetics of CT053PTSA.

In conclusion, CT053PTSA has potent antitumor and antiangiogenic activity in preclinical models. In this phase I, FIH clinical trial, CT053PTSA was generally well tolerated and safe for patients treated with the recommended dose. The results support the ongoing evaluation of the clinical activity of CT053PTSA in the treatment of cancer patients.

## Data availability statement

The datasets for this article are not publicly available due to concerns regarding participant/patient anonymity. Requests to access the datasets should be directed to the corresponding authors.

## Ethics statement

The studies involving human participants were reviewed and approved by Sun Yat-sen University Cancer Center Ethics Committee. The patients/participants provided their written informed consent to participate in this study. The animal study was reviewed and approved by Institutional Animal Care and Use Committee (IACUC) of HEC Pharma. Co. Ltd. Written informed consent was obtained from the individual(s) for the publication of any potentially identifiable images or data included in this article.

## Author contributions

H-YZ and LZ conceived and designed this study. Y-XM, F-RL and YZ collected and analysed the data, writing-original draft. NX and NK carried out the preclinical experiments. QC, Z-QC, Q-WL collected the clinical data. Y-XM, YH, Y-PY, W-FF enrolled patients. Y-LZ, QZ and Y-ZJ assisted the implementation of the clinical trial. All authors reviewed or revised the manuscript. All authors contributed to the article and approved the submitted version.

## Funding

This work was supported by National Nature Science Foundation of China (82073396 and 81872201 for H-YZ, 82002409 for Y-XM, 81872449 for LZ), Guangdong Basic and Applied Basic Research Foundation (2018A0303130243 for H-YZ, 2020A1515010020 for Y-XM), and HEC R&D Center, Sunshine Lake Pharma Co., Ltd.

## Acknowledgments

We thank all patients and their family for participating in the trial.

## Conflict of interest

Authors NX, NK, YLZ, QZ and YZJ are employed by Sunshine Lake Pharma Co., Ltd.

The remaining authors declare that the research was conducted in the absence of any commercial or financial relationships that could be construed as a potential conflict of interest.

## Publisher’s note

All claims expressed in this article are solely those of the authors and do not necessarily represent those of their affiliated organizations, or those of the publisher, the editors and the reviewers. Any product that may be evaluated in this article, or claim that may be made by its manufacturer, is not guaranteed or endorsed by the publisher.
