# Supplementary material for: Preclinical characterization and phase I clinical trial of CT053PTSA targets MET, AXL, and VEGFR2 in patients with advanced solid tumors
Source: Front Immunol. 2022 Oct 20;13:1024755. doi: 10.3389/fimmu.2022.1024755 (PMC9632963; doi:10.3389/fimmu.2022.1024755)
Supplement: Supplementary file 1 [file DataSheet_1.docx]

**Supplementary material and methods**

**Preclinical preparation**

For *in vitro* studies, CT053 (free-base form of CT053PTSA) was prepared in 100% DMSO at room temperature and diluted in relevant growth media. For *in vivo* studies, CT053 was formulated in a solution of 2% Hydroxy propyl methyl cellulose and 1% Tween 80.

**Biochemical kinase binding assay**

KinomeScan kinase binding assays were performed as described in reports i. Compound CT053 was screened against the panel at a single concentration of 10 μM in a primary screen, and results for primary screen binding interactions are reported as '% Ctrl', where lower numbers indicate stronger hits in the matrix.

%Ctrl Calculation: $[\frac{test compound signal-positive control signal}{negative control signal-positive control signal}$]🞩100

test compound = compound submitted by Calitor; negative control = DMSO (100%Ctrl); positive control = control compound (0%Ctrl).

**Kinase inhibition assay**

The inhibition profile of CT053 at 10 nM and 1 μM against a broad panel of 231 human kinase was determined using LANCE (Perkin-Elmer) or HTRF (Cisbio) technologies. Then the half maxmal inhibitory concentration (IC50) values were determined for those kinases in which at 1 μM more than 50% inhibition was observed. The IC50 values were determined by measuring phosphorylation of peptide substrate poly at ATP concentrations at the Km.

i Karaman MW, Herrgard S, Treiber DK, Gallant P, Atteridge CE, Campbell BT, et al. A quantitative analysis of kinase inhibitor selectivity. Nat Biotechnol. 2008;26(1):127-32. doi: 10.1038/nbt1358. PMID: 18183025.
