# Supplementary material for: Preclinical characterization and phase I clinical trial of CT053PTSA targets MET, AXL, and VEGFR2 in patients with advanced solid tumors
Source: Front Immunol. 2022 Oct 20;13:1024755. doi: 10.3389/fimmu.2022.1024755 (PMC9632963; doi:10.3389/fimmu.2022.1024755)
Supplement: Supplementary file 2 [file Table_1.docx]

**Supplementary Table 1.** *In vitro* kinase inhibition profile of CT053.

| **Kinase (h)** | **IC50 (nM)** |
| --- | --- |
| AXL | 3.4 |
| C-KIT | 960 |
| FLT3 | 8.6 |
| MERTK | 14 |
| MET | 68 |
| EphA4 | 370 |
| EphA6 | 81 |
| EphA8 | 620 |
| TXK | 270 |
| Tyro3 | 150 |
| Yes | 110 |
| VEGFR2 | 46 |
| FLT4 | 46 |
| FRK | 520 |
| HIPK4 | 180 |
| ITK | 240 |
| Fes | 48 |
| Fer | 330 |
| Fgr | 77 |
| FLT1 | 6100 |
| MNK2 | 55 |
| Musk | 310 |
| PDGFRα | 390 |
| RET | 200 |
| RON | 160 |
| LnyA | 240 |
| MLK | 160 |

**Supplementary Table 2.** Potent CT053 *in vitro* anti-proliferative activity correlates with MET mRNA level determined by RT-PCR in the 17 gastric tumor cell lines.

| **Cell line** | **Ratio of MET/GAPDH** | **IC50 (μM)** |
| --- | --- | --- |
| SNU-5 | 18.06 | 0.06 |
| SNU638 | 6.47 | 0.09 |
| SNU620 | 28.80 | 0.06 |
| GTL16 | 25.19 | 0.17 |
| MKN45 | 19.44 | 0.10 |
| HS746T | 5.64 | 0.14 |
| MKN74 | 0.35 | 6.88 |
| SNU-484 | 0.01 | 8.50 |
| SNU-16 | 1.38 | > 10 |
| AZ521 | 0.15 | > 10 |
| AGS | 0.61 | > 10 |
| NCI-N87 | 0.33 | > 10 |
| SNU-216 | 3.27 | >10 |
| SNU-601 | 2.14 | > 10 |
| KATOIII | 1.01 | > 10 |
| NUGC-4 | 2.95 | > 10 |
| NUGC3 | 1.03 | > 10 |

**Supplementary Table 3.** PK parameters of plasma CT053PTSA and CT053-M1 by cohort after a single dose (day 1) and multiple doses (day 28).

|  | Analyte | Dose (mg) | n | C_max_ | AUC_0-τ_ | AUC_0-168_ | AUC_0-inf_ | Cl/F | V_z_/F | T_max_ | T_1/2_ |
| --- | --- | --- | --- | --- | --- | --- | --- | --- | --- | --- | --- |
|  |  |  |  | (ng/mL) | (Hour*ng/mL) | (Hour*ng/mL) | (Hour*ng/mL) | (mL/Hour) | (mL) | (Hour) | (Hour) |
|  |  |  |  | Geometric Mean (CV% Geometric Mean) | | | | | | Median (Min, Max) | Mean ± SD |
| Single dose | CT053PTSA | 15 | 1 | 345 | 3760 | 6120 | 6190 | 2420 | 110000 | 3.0 | 31.4 |
|  |  | 30 | 1 | 796 | 12300 | 29100 | 29900 | 1000 | 46200 | 3.0 | 31.9 |
|  |  | 60 | 5 | 1340 (23.9) | 13700 (23.3) | 28700 (30.4) | 29200 (31.3) | 2060 (31.3) | 71800(25.9) | 2.0 (2.0, 3.0) | 24.7 ± 5.7 |
|  |  | 100 | 7 | 1890 (39.9) | 24400 (28.0) | 56000 (37.9) | 57700 (39.7) | 1730 (39.7) | 79800 (25.0) | 2.0 (2.0, 3.0) | 32.8 ± 8.5 |
|  |  | 150 | 4 | 2970 (42.7) | 30500 (19.4) | 65500 (24.8) | 68500 (29.2) | 2190 (29.2) | 99800 (16.8) | 2.5 (2.0, 3.0) | 33.9 ± 13.7 |
|  | CT053-M1 | 15 | 1 | 491 | 9500 | 27400 | 28400 | 529 | 24100 | 12.0 | 31.6 |
|  |  | 30 | 1 | 539 | 8300 | 46800 | 53800 | 558 | 41700 | 24.0 | 51.8 |
|  |  | 60 | 5 | 1180 (51.0) | 21200 (53.3) | 74800 (33.2) | 83400 (26.7) | 720 (26.7) | 45900 (59.7) | 24.0 (5.0, 24.1) | 46.0 ± 14.2 |
|  |  | 100 | 7 | 1190 (51.1) | 17400 (57.4) | 83000 (45.0) | 94200 (45.8) | 1060 (45.8) | 77500 (48.3) | 24.0 (24.0, 36.0) | 51.1 ± 7.9 |
|  |  | 150 | 4 | 2550 (148.4) | 43300 (154.8) | 180000 (85.8) | 207000 (68.4) | 726 (68.4) | 48000 (139.1) | 24.0 (12.0, 48.0) | 50.6 ± 27.8 |
| Multiple doses | CT053PTSA | 15 | 1 | 598 | 9510 | 23700 | 24000 | 1580 | 63500 | 2.0 | 27.9 |
|  |  | 30 | 1 | 1790 | 27600 | 81200 | 84100 | 1090 | 60200 | 2.0 | 38.4 |
|  |  | 60 | 3 | 2720 (31.3) | 37000 (23.2) | 89700 (33.4) | 91100 (34.7) | 1620 (23.2) | 60500 (14.8) | 2.0 (2.0, 2.3) | 26.1 ± 4.5 |
|  |  | 100 | 5 | 4330 (29.7) | 57300 (28.6) | 146000 (50.9) | 156000 (58.6) | 1740 (28.6) | 90900 (54.7) | 2.0 (2.0, 4.0) | 38.2 ± 14.3 |
|  |  | 150 | 1 | 2930 | 33600 | 50500 | 50600 | 4470 | 88700 | 2.0 | 13.8 |
|  | CT053-M1 | 15 | 1 | 992 | 22200 | 83400 | 98200 | 676 | 64200 | 3.0 | 65.9 |
|  |  | 30 | 1 | 2580 | 51600 | 160000 | 174000 | 582 | 45800 | 4.0 | 54.5 |
|  |  | 60 | 3 | 2820 (58.0) | 60800 (53.3) | 216000 (42.5) | 240000 (38.2) | 987 (53.3) | 66100 (101.7) | 5.0 (4.0, 24.0) | 48.2 ± 14.8 |
|  |  | 100 | 5 | 3560 (70.2) | 74200 (74.3) | 259000 (74.7) | 314000 (65.0) | 1350 (74.3) | 111000 (106.4) | 2.0 (2.0, 24.0) | 58.8 ± 18.6 |
|  |  | 150 | 1 | 11300 | 252000 | 662000 | 664000 | 595 | 18100 | 8 | 21.1 |

NOTE: The data were grouped on the basis of the dose level and study stage. Abbreviations: C_max_, peak concentration; AUC_0-24_, area under the concentration-time curve from 0 to 24 hours; AUC_0-168_, area under the concentration-time curve from 0 to 168 hours; AUC_0-inf_, area under the concentration-time curve from 0 to infinity; CL⁄F, oral clearance; Vz/F, apparent volume of distribution；T_max_, time to reach C_max_; T_1/2_, elimination half-life.

**Supplementary Table 4.** Circulation tumor DNA results of the patient after administration of CT053PTSA.

| **Gene** | **Mutation** | **Cycle 0** | **Cycle 1** | **Cycle 3** | **Cycle 6** |
| --- | --- | --- | --- | --- | --- |
| APC | p.E520X (c. G1558T) | 1.02% | 0 | 0 | 0 |
| AXL | p. A79T (c. G235A) | 1.55% | 0 | 0 | 0 |
| BRCA1 | p.E344X (c. G1030T) | 1.49% | 0.32% | 0.12% | 0 |
| EGFR | p. F712Y (c. T2135A) | 2.26% | 0.12% | 0 | 0 |
| FP300 | p.Q2332X (c.C6994T) | 1.96% | 0.19% | 0.21% | 0 |
| FLT3 | p.p893L (c.C2678T) | 1.75% | 0 | 0 | 0 |
| FLT4 | p.D998V (c.A2993T) | 1.62% | 0 | 0 | 0 |
| PALB2 | p.C800X (c.T2400A) | 1.19% | 0 | 0 | 0 |
